# Safe, mild ultraviolet‐B exposure: An essential human requirement for vitamin D and other vital bodily parameter adequacy: A review

**DOI:** 10.1111/phpp.12584

**Published:** 2020-08-09

**Authors:** J.L.M. Hawk

**Affiliations:** ^1^ Emeritus Professor of Dermatological Photobiology, Photodermatology Unit, St John’s Institute of Dermatology King’s College London London UK

**Keywords:** safe, mild UV exposure, vitamin D adequacy, other important bodily parameter adequacy

## Abstract

The enigma of skin sunburning, skin ageing and skin cancer and essential vitamin D production both resulting from solar ultraviolet‐B (280‐315 nm) (UVB) exposure has long puzzled photobiologists. Advice to patients by non‐photobiological clinicians is now often to sunbathe to acquire vitamin D adequacy. However, modern work shows only mild UVB exposure is needed to maintain satisfactory levels, which have been demonstrated as very similar in summer and winter from about 25° to 70° north. Even very careful high protection factor 15 sunscreen use does not prevent adequate production, although it is slightly reduced, such that obsessive use of very protective screens of 50 + might. Dark skin pigmentation too usually at most minimally impairs production. However, confinement indoors and widespread clothing cover can, but oral supplementation overcomes any such deficiency. Thus, vitamin D adequacy needs just mild regular UVB skin exposure well under sunburning levels, unlikely to cause significant skin damage. This suggests mild UVB exposure may also be needed for other bodily requirements, which is indeed so. Thus, it also prevents the development of contact dermatitis and polymorphic light eruption through suppressing adaptive immunity. It also prevents the occurrence of multiple skin infections resulting from this suppression through stimulating innate immunity and cutaneous bacterial defensin production. Finally, blood pressure is reduced through low‐dose UVB‐induced production of the vasodilator nitric oxide (though UVA, 315‐400 nm, is more efficient). Thus, mild UVB exposure is important for several aspects of internal health, whereas high‐dose exposure is extremely detrimental to cutaneous health.

Vitamin D, long of significant research and clinical interest, is now clearly a paradigm of the human need for minimal ultraviolet‐B (280‐315 nm) exposure, for a number of important health outcomes, as apparently only once briefly considered previously.^1^

Vitamin D is not in fact a vitamin, which is by definition an essential bodily substance only obtainable in the diet, but instead a steroid hormone naturally produced in the skin by solar UVB exposure in the skin sunburning, skin ageing and skin cancer‐producing wavelengths. It may also be obtained much less easily from the diet (particularly in cheese, fish, eggs and beef liver), if parathormone, calcitonin and dietary calcium are normal.^2^ It is also conveniently available as an oral supplement if natural measures are inadequate.

Such vitamin D is known to be absolutely necessary for bone health, particularly by virtue of its enhancement of calcium and phosphate absorption.^2^ However, in recent years, claimed rickets increases and questionably the occurrence of other major health issues from insufficiency have resulted from major sunlight avoidance to prevent darkening of the skin in some groups, photodermatoses in others, and particularly ultraviolet radiation‐induced skin cancer.^3^ In an effort to clarify this apparent conflict of evolutionary outcomes from exposure to the same radiation, this review now considers this and related matters in detail.

Figure 1 shows how 7‐dehydrocholesterol, a by‐product of bodily cholesterol formation, is converted via the formation of vitamin D_3_ to the active form, 1,25 (OH) vitamin D_3_. It should importantly be noted, however, that if ultraviolet exposure continues, the excess previtamin D_3_ produced is not directed into more vitamin D_3_ but instead by further ultraviolet conversion to the inactive isomers, lumisterol and tachysterol, thus not increasing vitamin D production. The conversion of previtamin D_3_ to vitamin D_3_ instead takes place by thermal conversion over several days in the skin environment, unaffected by any further ultraviolet exposure.

**FIGURE 1:**
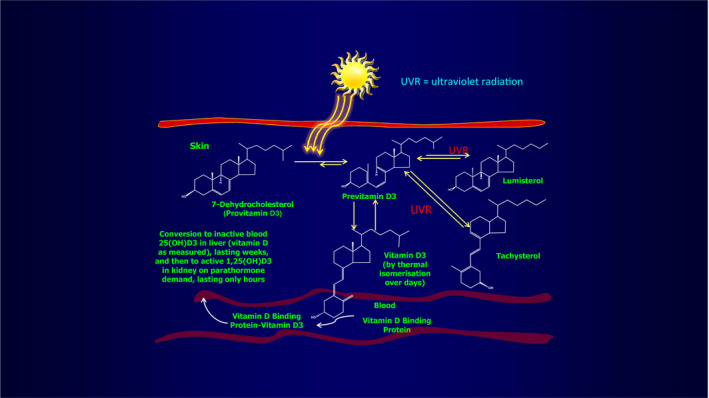
Ultraviolet radiation conversion of 7‐dehydrocholesterol to previtamin D3 before thermal conversion to vitamin D3 and chemical modification in the liver and kidney to 1,25 (OH) vitamin D3, the active form of the compound. Reprinted with permission from AAAS. From Holick et al, (1981) *J Invest Dermatol* 76:51‐58 ^4^

Since sunlight exposure has incontrovertibly been shown to induce the unpleasant, damaging, and sometimes fatal effects of skin sunburn, skin ageing and skin cancer, in addition to production of the essential vitamin D hormone, further investigation of the precise mechanisms of production of these different outcomes is necessary in an attempt to explain the clearly illogical conflict of evolutionary advantage and disadvantage.

This aim must inevitably involve consideration of the following issues. First, careful assessment of the exact wavelengths causing the genetic and related damage potentially leading to skin ageing and skin cancer, and those producing vitamin D, is needed to determine if they might perhaps be separable, just conceivably enabling the use of an appropriately filtered sunscreen.

Next, if the wavelengths producing both effects are similar, it is necessary to determine the amount of exposure needed to cause the unwanted genetic damage, and the amount required to maintain human vitamin D sufficiency, to assess if the amounts are significantly different.

Finally, it is necessary to determine the differential effect on genetic damage and vitamin D production, if any, of any form of photoprotection (whether from a life indoors, widespread clothing, sunscreen use, or particularly constitutional skin colour, as dark‐skinned races now live in weak solar ultraviolet radiation (UVR) areas), to see if these may contribute partly or completely to vitamin D deficiency.

Once these factors have been carefully assessed, as now to be discussed, a strategy to maintain vitamin D sufficiency without significant genetic and associated damage may conceivably be achievable.

Figure 2 shows that the solar wavelengths causing genetic damage and vitamin D production are somewhat different, the UVB region causing both, with the UVA segment also causing some skin damage but not producing vitamin D. However, these spectra are not sufficiently different for any refined sunscreen or other wavelength dissociation strategy to be effective at preventing genetic damage, while still enabling vitamin D production. Figure 2 also shows that 300nm radiation is optimal in producing vitamin D, confirmed artificially in practice with UVB lamps.^5^

**FIGURE 2:**
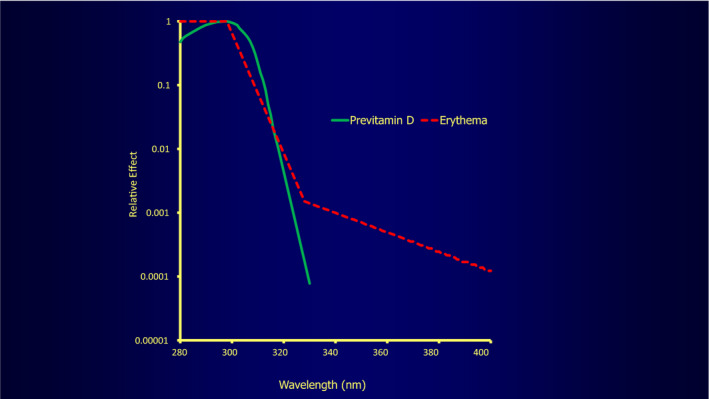
Commission Internationale de L’Éclairage (CIE) action spectra for production of sunburn erythema and previtamin D

Figure 3 demonstrates that high UVR exposure as often advocated by non‐photobiological clinicians for best vitamin D production is clearly effective at producing vitamin D but also much increases genetic damage and its associated skin sunburn, skin ageing and skin cancer risks.^6^ This however as shown too in Figure 2 is not the case for minimally exposed Danish skiers. In these subjects, increased vitamin D levels without significant thymine dimer production importantly suggest that the major clothing cover of skiers protects against skin damage while still permitting vitamin D production.^6^ Apart from in the mostly protected skiers, this situation of increasing genetic damage with only somewhat increasing vitamin D production is not ideal in evolutionary terms, so further consideration is necessary to decide what situation if any is ideal, as indeed foreshadowed by the situation in the skiers.

**FIGURE 3:**
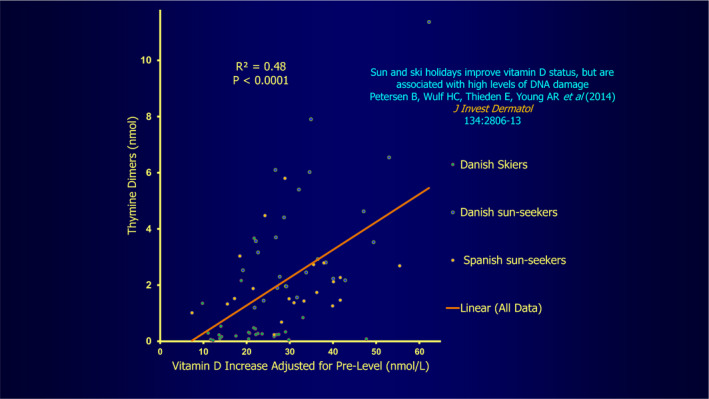
Correlation between DNA damage as assessed by thymine dimer production representing genetic damage and vitamin D levels. Reprinted with Permission from Datta P et al. (2012) *Photobiol Sci*. 11:1817‐1824. ^6^

In pursuit therefore of the aim of dissociating vitamin D production from significant skin damage, as seen in the just mentioned skiers, it has been shown that a mere half artificial UVB white skin sunburn dose (1.5 standard erythema doses, SEDs) every two to three days on four occasions only, just to areas about the size of the backs of the hands and face, or backs of hands and forearms, much increases vitamin D levels, clearly without major skin damage.^7^ Further, vitamin D production is no greater with higher doses or larger skin areas, while a quarter only of a sunburn dose to areas about the size of the backs of hands and face, or backs of hands and lower forearms gives some increase, clearly also without significant skin damage.^7^ Solar exposure though of the face, backs of hands and forearms is only effective from about April to September in Denmark, and maximal in early August.^5^ However, a small, artificial, winter UVB dose two‐weekly maintains summer vitamin D levels,^8^ if this is felt necessary, as systemic vitamin D levels diminish only slowly over winter before their spring boost, given the vitamin's storage in fat. Further, low vitamin D levels have been shown to go up rapidly with mild ultraviolet exposure, and high levels only a little with strong exposure,^9^, ^10^ as already suggested in Figure 1.

Telling other support for the suggestion that vitamin D homeostasis is maintained with just moderate ultraviolet exposure and not significantly increased with high exposure is shown by the fact that it is adequate under normal circumstances worldwide at latitudes with hugely varying solar intensities, in that mean vitamin D levels remain essentially similar from 25° to 70° North.^11^ They are therefore not apparently dependent on the availability of heavy sun exposure, being slightly higher in summer and lower in winter everywhere, at about 35‐100 nmol/L   (Figure 4). This is best explained overall by vitamin D having a long three‐month or so half‐life,^12^ with small regular exposures maintaining vitamin D levels, or else raising low vitamin D levels to normal rapidly, intense exposures doing this to only a minor amount more.^8^, ^9^

**FIGURE 4:**
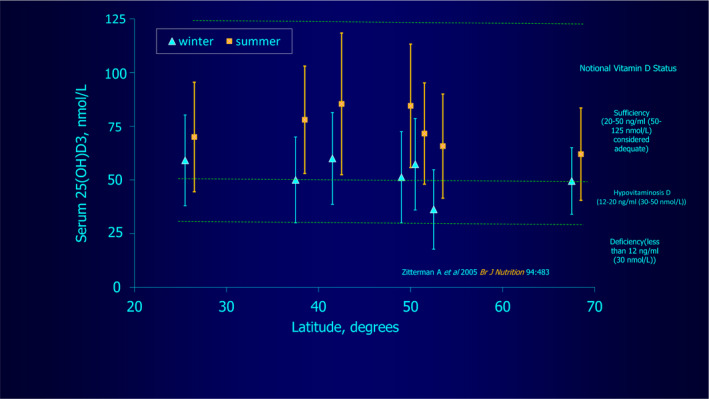
Plot showing vitamin D levels in summer and winter from 25^o^ ‐ 75^o^ latitude, showing very similar summer and winter average levels at all of these, from Table 3^11^, figure by kind courtesy of Professor Brian Diffey

Further strong evidence that marked sun exposure is not essential for adequate vitamin D production is that normal hair‐covered cows make sufficient amounts of the compound,^13^ with the response being proportionately reduced if the hide is partly covered, showing the area of exposed hide is responsible (not just the bare nose for example), as is also the case with the skin in man. Normal cows though do not suffer skin sunburn or cancers under their coats despite constant sun exposure,^14^ indicating adequate sun protection against those disorders while permitting sufficient vitamin D production, while human hair has also been shown to produce useful sun protection, varying between sun protection factors (SPFs) of 5 and 17, more if the hair is short (as in cows).^15^ This therefore provides yet further evidence that just mild regular solar exposure is all that is needed to maintain adequate vitamin D levels.

Vitamin D deficiency does of course occur and is greatest in the housebound, particularly in the older or disabled, in those in residential care, in subjects nearly fully covered by clothing, and in others who regularly avoid sun exposure or else work indoors.^16^ Constant extensive clothing cover leading to vitamin D deficiency is a particular risk in those with ethnocultural reasons for being protected in this way, even in heavily insolated Australia.^17^, ^18^

In addition, the effects of regular, careful sunscreen use have been of major concern as a possible significant cause of vitamin D deficiency. However, heavily sun‐exposed groups using a sun protection factor (SPF) 15 sunscreen in full, measured amounts in a cloudless March 2011 Tenerife study (28^o^N) developed adequate vitamin D increases.^19^ Such increases were in fact only a little less in sunscreen users than in a badly burned non‐sunscreen‐user cohort, with a broad‐spectrum sunscreen better for vitamin D production than just a UVB one. This is clearly because the former screen protected also against the less damaging UVA wavelengths, instead of just the maximal vitamin D producing UVB spectrum (Figure 5). This result somewhat resembles that shown in previous work, in which a white skin artificial UVB sunburning dose four times at two‐ to three‐day intervals to the whole body covered with SPF 8 sunscreen at different thicknesses generally maintained adequate vitamin D levels.^20^ However, the full recommended sunscreen thickness of 2 mg/cm^2^ led to no increase, almost certainly because the UVB was very efficiently absorbed by the sunscreen used. Further proof that sunscreen application only mildly reduces vitamin D production is that double applications in another study before solar exposure for more reliable, overall skin protection had no more effect on reducing vitamin D production than just one application.^21^

**FIGURE 5:**
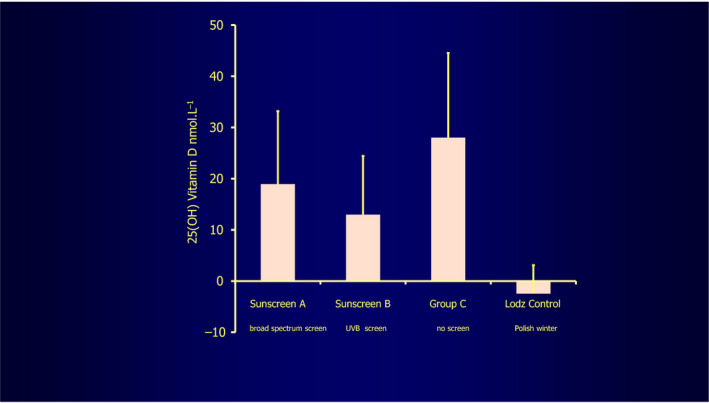
This shows the effect of very carefully applied (under supervision) sunscreens of high sun protection factor 15, A against UVB and UVA, B just against UVB, compared with C, no sunscreen protection. The sunscreens only mildly reduce vitamin D production, sunscreen A less so. The Łódź control plot refers to control volunteers remaining in the Łódź Polish winter^19^, figure by kind courtesy of Professor Antony Young

Given that some reduction in vitamin D production did occur with an SPF 15 sunscreen during heavy ultraviolet exposure in Tenerife, as described above, it does seem very likely that obsessively careful, very high SPF sunscreen use, say 50+, would prevent solar vitamin D production. However, non‐obsessional use as generally occurs in normal subjects achieves only a third or often less of the SPF, strongly suggesting that significantly reduced production does not often happen, even SPF 50 seeming safe.^22^

Further, and perhaps unexpectedly, even marked skin pigmentation generally appears as shown by two separate research groups to have at most a minor effect on ultraviolet radiation‐induced vitamin D production.^9^, ^23^ This would appear likely to be because the cholesterol‐derived vitamin D precursor is present in high enough concentrations superficially to permit vitamin D production, especially as the melanin is concentrated near the epidermal basal layer (Figure 6). However, Kift et al^24^ have questioned this amongst South Asians in Manchester, UK, their deficiency very possibly occurring because pigment genes can at times have a greater effect than pigmentation in determining UVB‐induced vitamin D production.^25^ In addition, careful sun protection to avoid deeper than constitutional skin tanning and also dietary differences in the Manchester group may partly contribute.

**FIGURE 6:**
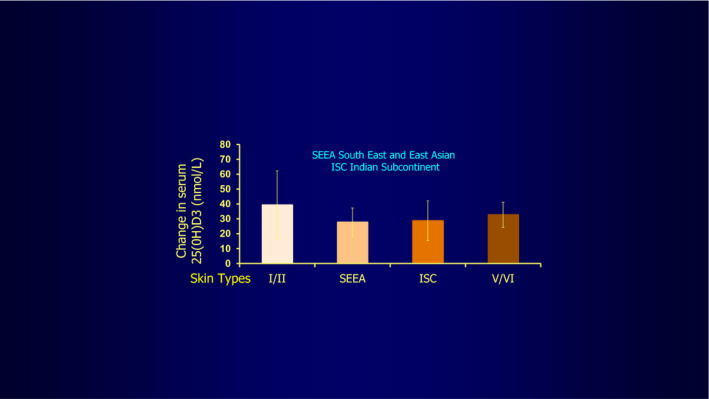
This shows the minor differences in vitamin D production in the volunteer subjects tested, of skin types I to VI, irradiated all over with solar simulated radiation, apart from under their underwear, indicating skin pigmentation does not inhibit production to a significant degree^23^, figure by kind courtesy of Professor Antony Young, but also shown by Bogh et al^9^, though pigment gene differences can also play a part (Datta et al^25^)

In overall summary, therefore, higher average summer and lower average winter vitamin D levels worldwide are similar everywhere, and only mild, regular ultraviolet‐B exposure seems necessary to maintain these satisfactory levels. This is largely because low vitamin D levels go up rapidly with mild ultraviolet exposure, while high levels go up only a little, even with strong, regular exposures. Thus, just half a sunburn dose for white skins regularly to areas about the size of the backs of hands and face or lower forearms, and merely a quarter of a sunburn dose on areas about the size of the backs of hands and face or lower forearms, are sufficient to raise levels. Pigmentation, even if dark, animal coats and normal, even high SPF sunscreen use, or double sunscreen applications before exposure, have at most a mild reducing effect on vitamin D production and circulating levels. On the other hand, low vitamin D levels are likely to result from major intentional sun avoidance, constant major clothing cover, major confinement indoors, obsessive high SPF sunscreen use, an inappropriate diet to some extent or a combination. Thus, just small regular exposures appear to be needed for vitamin D production in normal subjects with no specific exposure time needing or even able to be advocated, as solar ultraviolet levels vary hugely and cannot be prejudged.

If in spite of this, concerns about vitamin D adequacy persist, a safe and effective alternative, even in the presence of normal, careful sun exposure, is the administration of oral vitamin D. Recommended doses for this should be 600 IU/d (15 μg) in adults but 800 IU/d (20 μg) in subjects over 70 years of age, with a safety maximum of 4000 IU/d (100 μg).^26^ However, such medication is not recommended in patients with hypercalcaemia, hypervitaminosis D or renal osteodystrophy with hyperphosphataemia, and preferably not either in atherosclerosis, impaired cardiac or renal function, vitamin D sensitivity or sarcoidosis, when mild sun exposure is in fact preferred.^27^

This overall vitamin D sun exposure situation seems best to fit with a putative mammalian need, briefly considered previously,^1^ for minimal, largely harmless, regular sun exposure as an evolutionary advantage, not a drawback, in several ways, as below.

First, it avoids constant delayed type hypersensitivity reactions inducing the sun rash, polymorphic light eruption, as supported by the fact that very low doses of UVB phototherapy are needed to give months of relief from the condition,^28^ and that immunological tolerance or “hardening” frequently develops during the summer. It also avoids constant allergic contact dermatitis reactions to the known huge number of environmental allergens, both of these reactions occurring through the ultraviolet‐induced suppression of adaptive immunity.^29^, ^30^, ^31^

Secondly, it induces bactericidal defensins by activating innate immunity, to prevent skin infection likely with the ultraviolet‐reduced adaptive immunity just mentioned.^29^, ^30^, ^31^

Thirdly, it somewhat helps reduce blood pressure, although UVA is more efficient at this,^32^ reducing the likelihood of myocardial infarctions and cerebrovascular accidents, by increasing cutaneous production of the vasodilator, nitric oxide.^33^

And finally, it avoids vitamin D deficiency, as fully discussed in this review.

In addition, as if to confirm the need for only minimal human UVR exposure, excessive ambient UVR produces an unpleasant warning through the eye leading sufferers to shun such exposure by seeking shade or going indoors, unless wearing sunglasses.^34^

Therefore, a safe and convenient strategy for maintaining adequate vitamin D status and receiving the other advantages mentioned above would seem to be to live normally, using photoprotection as suggested to minimize skin ageing and cancer risks, but if in at risk group, take vitamin D supplementation.

The exact, putative amount and frequency of low‐dose ultraviolet exposure needed to achieve these very useful aims without significant genetic damage remains to be assessed, and this matter should now be addressed as a follow‐up to the items addressed in this review.

## CONFLICT OF INTEREST

None declared.
